# Multiattention Mechanism 3D Object Detection Algorithm Based on RGB and LiDAR Fusion for Intelligent Driving

**DOI:** 10.3390/s23218732

**Published:** 2023-10-26

**Authors:** Xiucai Zhang, Lei He, Junyi Chen, Baoyun Wang, Yuhai Wang, Yuanle Zhou

**Affiliations:** State Key Laboratory of Automotive Simulation and Control, Jilin University, Changchun 130022, China; zhangxc21@mails.jlu.edu.cn (X.Z.); cjy22@mails.jlu.edu.cn (J.C.); wangby22@mails.jlu.edu.cn (B.W.); wangyuhai@jlu.edu.cn (Y.W.); zhouyl22@mails.jlu.edu.cn (Y.Z.)

**Keywords:** multimodal fusion, attention mechanism, 3D target detection, deep learning

## Abstract

This paper proposes a multimodal fusion 3D target detection algorithm based on the attention mechanism to improve the performance of 3D target detection. The algorithm utilizes point cloud data and information from the camera. For image feature extraction, the ResNet50 + FPN architecture extracts features at four levels. Point cloud feature extraction employs the voxel method and FCN to extract point and voxel features. The fusion of image and point cloud features is achieved through regional point fusion and voxel fusion methods. After information fusion, the Coordinate and SimAM attention mechanisms extract fusion features at a deep level. The algorithm’s performance is evaluated using the DAIR-V2X dataset. The results show that compared to the Part-A2 algorithm; the proposed algorithm improves the mAP value by 7.9% in the BEV view and 7.8% in the 3D view at IOU = 0.5 (cars) and IOU = 0.25 (pedestrians and cyclists). At IOU = 0.7 (cars) and IOU = 0.5 (pedestrians and cyclists), the mAP value of the SECOND algorithm is improved by 5.4% in the BEV view and 4.3% in the 3D view, compared to other comparison algorithms.

## 1. Introduction

With the continuous development of intelligent driving technology, the requirements for environmental awareness performance are constantly improving. Therefore, 3D object detection has received great attention [1,2,3]. However, single-modal 3D object detection alone cannot handle such a complex scene. Therefore, the research focuses on multimodal three-dimensional object detection, a key method to improve the environment perception of intelligent driving.

Convolutional Neural Network (CNN)-based techniques have performed well on detection datasets of images [4]. Significant achievements have been made in 2D target detection in images [4,5,6,7]. However, these methods cannot be directly applied to 3D detection due to the different input modes. Compared to images, LiDAR can precisely locate objects in 3D space, while 3D point clouds can provide detailed geometry and capture the 3D structure of the scene. Detection techniques based on LiDAR data are generally superior to camera-based 3D detection techniques [4,5,6,7,8,9]. Point clouds, conversely, are irregular and cannot be directly processed by powerful deep learning models, such as convolutional neural networks. This presents an excellent challenge for effective feature learning. Some of these methods convert 3D point clouds into depth maps and bird’s-eye-view (BEV) maps by manual processing and then process them in a 2D CNN manner for vehicle detection and classification [8]. However, the manually extracted features must fully utilize the information from the point cloud and may lead to performance degradation when detecting fewer points or variable geometry objects. There are also approaches [9] that use a 2D detector to generate a 2D detection frame on the image, transform the 2D detection frame into a proposed region in 3D space, and then use the PointNet [10] architecture for target detection on the point cloud. However, this approach relies heavily on the performance of the 2D target detector and cannot take advantage of the 3D information to generate robust bounding boxes.

Recent research in 3D target detection has primarily focused on utilizing end-to-end trainable neural networks that can directly process point cloud data without requiring manual feature extraction, as seen in the case of BEV maps. Vote3D [11] uses a sliding window on the sparse volume of a three-dimensional voxel grid to detect objects. The hand-crafted geometric features are extracted on each volume and fed into the SVM classifier [12]. Vote3Deep [13] also uses voxel representations of point clouds but uses 3D convolutional neural networks to extract the features of each individual [14]. The main problem with voxel representation is efficiency, as 3D voxel grids typically have high dimensions. In contrast, VeloFCN [15] projects a 3D point cloud onto the front view to obtain a 2D depth map. The vehicle is then detected using a 2D CNN on the depth map. Qi et al. [16] developed a neural network architecture that directly utilizes point clouds as input and output class labels. This allows for the learning of representations from raw data. However, this approach cannot be applied to target detection and localization due to limitations within the network architecture and the high storage cost. Zhou and Tuzel [17] proposed VoxelNet, which involves voxelizing the point cloud and employing a series of voxel feature encoding (VFE) layers to overcome this issue. This processing allows the VoxelNet network to directly extract the point cloud within the voxel using 3D convolution. Another model proposed by S. Shi et al., PointRCNN [14], offers superior performance in 3D target detection of point clouds compared to the two-stage image target detection network, Fast-RCNN [18]. PointRCNN requires further clarification regarding the point cloud pooling strategy despite its advantages. The pooling of different proposals may lead to pooling the same set of points, resulting in the loss of geometric information encoding. To address this, a new point cloud pooling operation for regions of interest, Part-A2 [19] networks, was proposed, which retains all information from nonempty and empty voxels within the proposals, eliminating ambiguity from previous point cloud pooling strategies. Although these methods have demonstrated improved performance, they all rely solely on a single modality, namely point cloud data. RGB images, on the other hand, offer denser texture, color, and additional information than point clouds, suggesting that both modalities can be leveraged to enhance detection performance.

**Figure 1:**
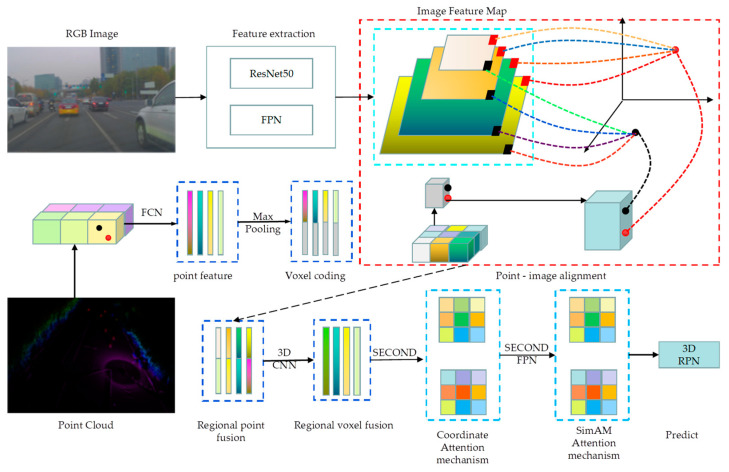
The framework of 3D object detection based on multimodal fusion. After the image and point cloud data pass through their respective feature extraction networks, the image and point cloud features are obtained, respectively. Then, the point cloud and image features are matched, and the multimodal information fusion operation is achieved by regional point fusion and voxel fusion. Finally, 3D target prediction output is produced using the Coordinate and SimAM attention mechanisms.

This paper aims to solve the abovementioned issues by implementing a fusion method that combines LiDAR point cloud and RGB image for 3D target detection. The process involves utilizing ResNet50 as the backbone network for image feature extraction and the FPN structure to gather multilevel features from the images. Point cloud and image early and late fusion are achieved through regional point fusion and voxel fusion. The Coordinate and SimAM attention mechanisms further process the information extracted after point cloud image fusion. Finally, features are outputted using the SECOND-FPN structure. The network structure framework depicted in Figure 1 demonstrates the approach proposed in this paper.

## 2. Related Work

This section provides an overview of 3D object detection techniques for vehicles, pedestrians, and cyclists in the context of autonomous driving and ADAS systems. The research in this field has seen significant advancements in recent years. It can be categorized into three main approaches: camera-based sensor, LiDAR sensor, and camera and LiDAR multimode fusion.

### 2.1. Target Detection Based on Camera Sensor

Various methods have been developed to estimate 3D bounding boxes using 2D image information [20,21,22,23,24]. For example, geometric constraints between three-dimensional and two-dimensional boundary boxes are used to restore the pose of three-dimensional objects in object detection [25,26]. Similarly, refs. [27,28,29] leverage the similarity between 3D objects and CAD models to estimate the attitude of 3D object detection. Chen et al. [30,31] propose an energy function to represent the three-dimensional geometric framework and score predefined 3D boxes. Recent studies [32,33] have also explored using stereo images to enhance the performance of 3D object detection in stereoscopic cameras. However, these methods often need more precise depth information, resulting in coarse 3D detection results that are susceptible to changes in appearance.

### 2.2. Target Detection Based on LiDAR Sensor

The research on the use of LiDAR for three-dimensional target detection has attracted widespread attention. The manual feature method was initially successfully adopted [34,35,36,37,38], but only in scenes with clear texture information and comprehensive 3D data. Subsequently, voxel mesh occupation is introduced to represent three-dimensional point clouds [11,13,39], and three-dimensional bounding box calculation is realized by three-dimensional convolution. However, these methods are computationally and memory-intensive. To address this problem, a method based on the BEV (Bird’s-Eye-View) feature map is proposed [40,41]. This method assumes that the point cloud has a sparse vertical height, which may not hold in most cases.

Another approach to 3D object detection is to use a two-stage object detection network. In the first phase, region proposals are generated. Then, in the second phase, point clouds and associated semantic features within the candidate region are utilized to improve the accuracy of the 3D bounding box. Some methods utilize sophisticated 2D detectors to obtain the corresponding image’s two-dimensional region of interest (ROI). Inverse projection transforms these regions into three-dimensional spaces, forming a cone-shaped three-dimensional point cloud [9,42]. Finally, using these conical 3D point clouds as input, PointNet/ConvNet extracts features of interest within the area.

### 2.3. Target Detection Based on Camera and LiDAR Fusion

The existing research on multimode information fusion for target detection using LiDAR and RGB image data is limited [42,43]. To address the challenge of effectively fusing multiview features, Zhang et al. [44] proposed a multiview feature adaptive fusion framework for 3D object detection. Chen et al. [1] introduced the Multi-View 3D Target Detection network (MV3D), which takes in LiDAR and image data and combines regional features to generate 3D bounding boxes. While this method achieves promising results through multimode fusion, it suffers from issues, such as point cloud information loss and late-stage fusion of multimode information, which restrict the exchange of information between different data modes. Ku et al. [45] proposed a multimode fusion network incorporating regional features to overcome these limitations. By designing RPN structures with high-resolution feature mapping, this network achieves better detection results and improves its performance in detecting and classifying small objects.

In a different approach, Qi et al. proposed Frustum PointNets [46], a 3D target detection method that integrates LiDAR and image data. This model initially employs a 2D detector to generate a 2D detection box on the image, which is then converted into a proposed region in 3D space. Finally, the PointNet architecture performs target detection on the point cloud. However, this approach primarily focuses on utilizing image information and only partially exploits the potential of both data sources [46]. We adopted an attention-based multimode fusion strategy to address this limitation and enhance the exchange of information between multiple modes at the early stages.

## 3. Proposed Method

### 3.1. Image Feature Extraction Framework

**Figure 2:**
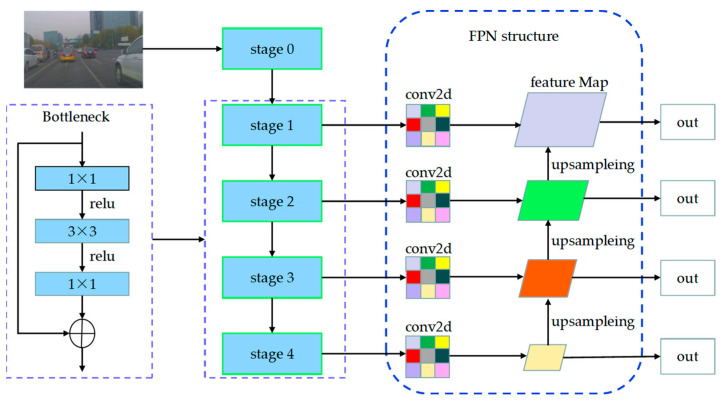
Image feature extraction network framework. After passing through four bottleneck modules of ResNet50, image data are exported with four levels of feature graphs. The feature graphs at four levels are collected for feature stitching, and feature output is performed from four dimensions.

In image processing, the performance of convolutional neural networks improves with increasing depth to extract more advanced features. However, traditional convolutional neural networks will encounter problems, such as network decay, gradient explosion, and gradient disappearing with increasing depth, resulting in a decline in network performance with increasing depth. To overcome these problems, this paper adopted ResNet50 [47], which employs a residual module as the backbone network for image extraction (shown in Figure 2). The residual module’s introduction solves the network recession issue, while adding a BN layer addresses gradient disappearance and explosion. ResNet50 consists of five stages, namely Stage 0 to Stage 5. The initial stage performs more straightforward tasks, primarily preprocessing the input data. The subsequent four stages (stage 1 to stage 4) comprise bottleneck structures that extract high-level semantic features from the image.

To enhance the ability to integrate low-level details and high-level semantics, it is necessary to expand the sensory field of the bottom layer and improve the detection performance of small targets. This paper adopted the multiscale feature pyramid structure of the FPN in order to fuse the low-level detail information and the high-level semantic information, thereby increasing the sensory field of the bottom layer and enhancing the detection performance of small targets. The FPN structure, as depicted in Figure 2, consists of three lines: the self-low-upward, the self-top-downward, and the lateral link. The low-up module continuously pools the forward propagation feature maps, resulting in four feature maps of different sizes. The top-down module upsamples the small-size feature maps and performs splicing and fusion operations with the prominent feature maps from the low-up process. The horizontal link adjusts the output of different feature maps using a 1 × 1 convolutional kernel with 256 channels, facilitating subsequent fusion. Finally, the final prediction output is performed on the four feature maps, forming a multilevel and multiscale feature pyramid structure.

### 3.2. Point Cloud Feature Extraction

#### 3.2.1. Point Cloud Voxelization

**Figure 3:**
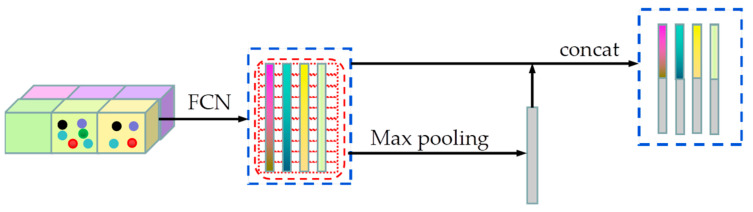
Point cloud voxelization and feature extraction. The feature extraction of each point in each voxel is carried out. Then, the maximum pooling operation is carried out on the horizontal channel to obtain the feature as the voxel feature. Combining the most point feature and the voxel feature represents the final feature.

In order to facilitate feature extraction, this paper adopted a method similar to VoxelNet [17] to perform voxelization, grouping, and random extraction operations on the sparse point cloud throughout the space. Voxelization divides the 3D space into equally spaced voxels, with voxel sizes defined based on the range of the point cloud along the Z, Y, and X directions denoted as D, H, and W, respectively. Points are then grouped based on the voxel where they are located. Due to factors such as distance, occlusion, and sparsity, the number of points in each voxel can vary, as shown in the leftmost voxelization of Figure 3.

In this paper, we address the issue of a memory burden on the computational platform that arises from processing the high-resolution LiDAR point cloud, which consists of millions of points. Directly processing these point clouds can overwhelm the computational platform due to the high number of points. Another challenge is the variable density of the point cloud throughout the spatial height, which can impact the accuracy of the detection results. We propose randomly extracting a fixed number of point clouds in each voxel to mitigate these issues. This strategy reduces the memory burden on the computational platform and helps balance the voxel distribution, resulting in improved training diversification.

#### 3.2.2. Point Cloud Feature Extraction

In the point cloud feature extraction network, which is shown in Figure 3, first $p_{i}={\left[x_{i},y_{i},z_{i},r_{i}\right]}^{T}$ represents the coordinates of each point in the voxel. The voxel has four elements that represent the X, Y, and Z coordinates and reflectance. Before feature extraction, the initial feature of each point cloud is represented by the point coordinates and the center position relative to the point coordinates. This initial feature can be expressed as $p_{i}={\left[x_{i},y_{i},z_{i},r_{i},x_{i}-v_{x},y_{i}-v_{y},z_{i}-v_{z}\right]}^{T}$, where $v_{x},v_{y},v_{z}$ denotes the center position coordinates of voxels [44]. The FCN feature extraction network then extracts the features of the points inside each voxel. After the feature extraction of each point inside the voxel is completed, the features are extracted as the voxel features using the maximum pooling method in the channel corresponding to each point. Finally, each voxel’s extracted point features and voxel features are spliced together as the final features. The FCN network comprises a linear layer, a batch normalization (BN) layer, and an activation function layer (ReLU). All the nonempty voxels are encoded in the same form and share all the parameter sets in the FCN network. The FCN structure transforms the input point cloud data into high-dimensional features. This structure encodes point interactions within voxels, allowing the final feature representation to learn to describe shape information. Therefore, point cloud features are extracted by stacking three layers of this structure in a point cloud feature extraction network.

### 3.3. Multimodal Fusion

This paper proposes two fusion techniques to improve the performance of 3D target detection in infrastructure view by extending the VoxelNet framework to fuse point cloud and image data. As mentioned, the VoxelNet [17] model is based on a single modality. However, this study enhances it by adding a multimodal fusion scheme, improving the network’s performance.

#### 3.3.1. Regional Point Fusion

**Figure 4:**
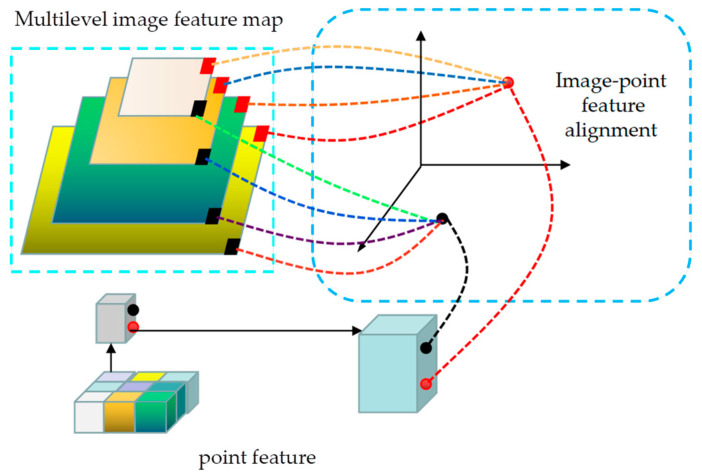
The framework of regional point fusion. The point features are transformed into four-level image features utilizing coordinate transformation, and then the point features and image features are aligned.

This early fusion technique utilizes image features to aggregate 3D point clouds, enhancing the contextual information, as illustrated in Figure 4. The method initially employs ResNet50 and FPN structure extraction networks to extract high-level feature maps from images with multilevel semantic encoding. These feature maps are then utilized to project each 3D point onto different layers’ feature maps using a calibration matrix, thereby identifying the corresponding position on the image for each point. Subsequently, a 3 × 3 convolution is applied to extract centralized features of the small region associated with each point. These features are combined with the point cloud features derived from the previous feature extraction network. Following the splicing of features, a set of FCN layer network structures is employed for further processing. Ultimately, these processed features are employed in the subsequent detection stage.

The advantage of this approach is the ability to connect multilevel image features to point cloud features at an early stage. This provides information on the location of the image corresponding to each point and feature within a small area of that point. Subsequently, the network can learn helpful information in both modalities through the FCN layer.

#### 3.3.2. Regional Voxel Fusion

Region voxel fusion employs a relatively late fusion strategy compared to region point fusion features. After the 3D convolution has extracted the features, the 3D space is transformed into a 2D space. Subsequently, an expansion convolution with an expansion coefficient of 1 is used to further the fusion of a more extensive range of information to increase the sensory field. After region point fusion, semantic features of the image are attached to the voxel level. Each voxel contains both point cloud and image features within it. In order to fully consider the information interaction between voxel contexts, this paper adopted three 3D convolutions for the fusion and extraction of regional features on 3D information.

Regional voxel fusion is a relatively late fusion strategy but offers certain advantages. Firstly, this approach enables the fusion of features projected from the image onto the point cloud at the voxel level, thereby enhancing the combination of feature information in multiple regions near the point cloud. Secondly, expanding convolution can enhance the receptive field and facilitate the detection of smaller objects.

### 3.4. Fusion Feature Extraction and Attention Mechanism

#### 3.4.1. Coordinate Attention Mechanism

**Figure 5:**
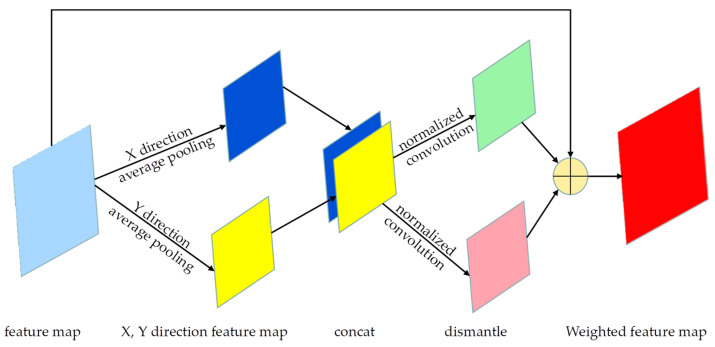
The schematic diagram of coordinated attention mechanism. The feature map is average pooled in the X and Y directions. The features in the two directions are spliced, different convolution is used to extract the features, and, finally, the feature addition is used to output.

After fusing point cloud and image information, this paper utilized the Coordinate attention mechanism for enhancing the feature map processing, as depicted in Figure 5.

This paper proposes an attention mechanism that considers both channel aspect information and position information in the feature map’s horizontal and vertical dimensions. To ensure that spatial information is not compressed into the channel and to enable spatial interaction of the captured information, average pooling is performed separately in the *X*-axis and *Y*-axis directions. The pooled results are then concatenated and subjected to convolution operation, allowing interactions between the positions in the two axes. Once the information interaction is completed, the weights are sparsely computed along the two axes, resulting in a final feature that retains positional- and channel-specific information.

#### 3.4.2. SimAM Attention Mechanism

**Figure 6:**
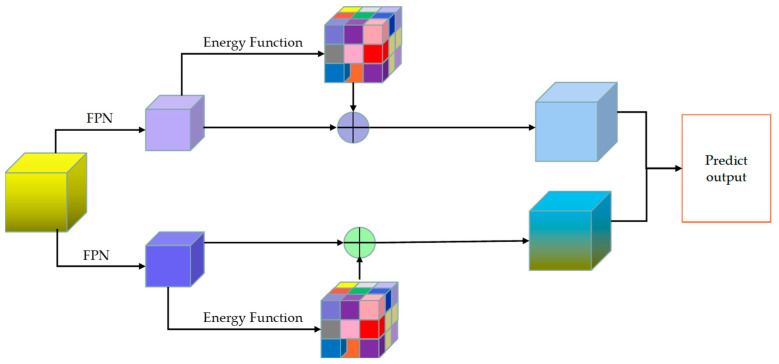
The schematic diagram of SimAM attention mechanism. The feature map uses two FPN structures to extract their features, takes the minimum energy function as the goal to extract their respective weight coefficients, and then weights the features extracted by FPN. Finally, the two weighted features are spliced and output.

After completing the fusion feature extraction with the Coordinate attention mechanism, this paper focused on utilizing the FPN structure in the point cloud for two-level feature extraction. This approach ensures that the deeper-level feature map contains richer semantic information, while the shallow-level feature map preserves more complete geometric details. Figure 6 demonstrates the SimAM parameter-free attention mechanism employed in this study to assess the significance of each neuron in the network. This mechanism accomplishes the differentiation by defining the energy function’s form, where neurons with higher energy functions are assigned greater weights due to their increased importance. In comparison, those with lower energy functions are assigned lower weights.

## 4. Loss Functions

This paper firstly parameterized the 3D truth frame as follows:$\left(x_{c}^{g},y_{c}^{g},z_{c}^{g},l^{g},w^{g},h^{g},\theta^{g}\right)$. $x_{c}^{g},y_{c}^{g},z_{c}^{g}$ represent the center of the 3D truth frame, $l^{g},\omega^{g},h^{g}$ represent the length, width, and height of the 3D truth frame, and $\theta^{g}$ represents the rotation angle along the *Z*-axis. At the same time, the paper parameterized the Anchor designed by ourselves in the target detection as $\left(x_{c}^{a},y_{c}^{a},z_{c}^{a},l^{a},w^{a},h^{a},\theta^{a}\right)$ and defined seven residual coefficients of regression. These coefficients represent the offset relative to the center coordinate, the elongation or shortening ratio of the three dimensions of length, width, and height, as well as the deviation around the direction of the *Z*-axis. The calculations of these seven coefficients are shown as follows.

(1) $$\begin{array}{l} \Delta x=\frac{x_{c}^{g}-x_{c}^{a}}{d^{a}},\Delta y=\frac{y_{c}^{g}-y_{c}^{a}}{d^{a}},\Delta z=\frac{z_{c}^{g}-z_{c}^{a}}{h^{a}},\\ \Delta l=\log(\frac{l^{g}}{l^{a}}),\Delta w=\log(\frac{w^{g}}{w^{a}}),\Delta h=\log(\frac{h^{g}}{h^{a}}),\\ \Delta \theta =\theta^{g}-\theta^{a} \end{array}$$

In the designed anchor frame, Equation (1) $l^{a}$ represents the diagonal length of the 3D frame base. In order to estimate the oriented 3D detection frame directly, Δx, Δy, and the diagonal of the 3D frame $d^{a}$ were normalized. This differs from the method provided by Li et al. [15]. Finally, the loss function is defined in this paper, as shown below.

(2) $$\begin{array}{c} L=\alpha \frac{1}{N_{pos}}\sum_{i}L_{cls}(p_{i}^{pos},1)+\beta \frac{1}{N_{neg}}\sum_{j}L_{cls}(p_{j}^{neg},0)+\\ \frac{1}{N_{pos}}\sum_{i}L_{reg}(u_{i},u_{i}^{*}) \end{array}$$

In Equation (2), $N_{pos}$ represents the number of positive anchor frames and $N_{neg}$ represents the number of negative anchor frames. $p_{i}^{pos}$ represents the probability that the ith anchor frame is predicted to be a true label, and $p_{j}^{neg}$ represents the probability that the j anchor frame is predicted to be a false label. $u_{i}$ represents the bounding box regression parameter of the ith anchor box, and $u_{i}^{*}$ represents the bounding box regression parameter of the ith anchor box corresponding to the truth box. $L_{cls}$ denotes cross-entropy loss, α, β denote balanced positive and negative sample parameters, and $L_{reg}$ denotes regression parameters.

## 5. Experimental Section and Discussion

### 5.1. Datasets

This paper proposes a multimodal fusion with multiple attention mechanisms for a 3D target detection algorithm. The algorithm will be evaluated on the DAIR-V2X dataset, which contains 15,285 image data and 15,285 frames of point cloud data. The dataset is further decomposed into a training set and a validation set in the ratio of 7:3. After the split, the training set consists of 10,700 samples, and the validation set consists of 4585. The evaluation will analyze the effectiveness of the proposed multimodal approach by comparing it with previously published methods for 3D target detection tasks. The evaluation will consider three difficulty levels, easy, medium, and hard, based on object size, visibility (occlusion), and truncation.

### 5.2. Data Enhancement

This study aims to solve the overfitting problem in the network training process by enhancing the data of both images and point clouds. We performed the following steps on the image: First, we resized the image to two different sizes, (640,192) and (2,560,768). Next, we used [102.42,117.36,124.58] as the mean and [1.0,1.0,1.0] as the regularized variance. Finally, we flipped the image horizontally with a flip ratio of 0.5. We flipped within the specified angle range for point clouds and zoomed in the [0.90,1.10] range. Like the image, the point cloud was flipped horizontally with a flip ratio of 0.5.

In addition, all ground truth boxes (bi) and the entire point cloud (M) were scaled globally. Specifically, we multiplied the XYZ coordinate and three-dimensional space of each bi and the XYZ coordinate of a point in M by a random variable evenly distributed in [0.95,1.05]. In addition, this paper introduces global scaling in image-based classification [48] and detection tasks [18] to enhance the network’s ability to detect targets of different sizes and distances, thereby improving overall robustness.

### 5.3. Experimental Seting

We conducted experiments on the 3D detection using the Pytorch framework in Ubuntu 18.04. The experiments were implemented on a computer with the following specifications: 1 NVIDIA RX3060 graphics card with 12 GB GPU memory, i9-7900X @3.60 GHz × 10 processors, and 64GB RAM. We used Adam with hyperparameters for training optimization and set to 0.9 and 0.999. The number of iterations was set to 500 K, with an initial learning rate of 0.0001. The learning rate decayed every 20 epochs with a decay rate of 0.9. Due to memory constraints, we conducted the experiments in four batches to obtain better experimental results with the available hardware, as larger batch sizes usually lead to more improvements. It is important to note that background should not be ignored in training and testing processes.

### 5.4. Experimental Parameters

#### 5.4.1. Image Detection Networks

In this study, ResNet50 + FPN architecture was used for feature extraction in image object detection. The training dataset was the DAIR-V2X dataset, and data augmentation techniques were applied. During training, the shortest edge of the image was rescaled to 600 pixels. This paper used four scale anchors {4,8,16,32} and three aspect ratios {0.5,1,2} for the final output layer. If the intersection of the anchor point with the ground truth bounding box exceeded 0.7, the anchor point was marked as positive, and if the IOU was less than 0.3, the anchor point was marked as negative. The network was trained by stochastic gradient descent with a learning rate 0.0005 and a momentum of 0.9. In the multimodal fusion training process, once the image detection network training was completed, the parameters of the image network were frozen, and the weight coefficient of the image backbone network remained unchanged.

#### 5.4.2. Point Cloud Detection Networks

This research focuses on point cloud detection using VoxelNet architecture. The point cloud was considered along the Z, Y, and X axes of [−3,1] × [−40,40] × [0,70.4] meters, respectively. To ensure accuracy, points projected outside the image boundaries were removed [25]. For voxel size, we chose vD = 0.05, vH = 0.05, vW = 0.1 m, and we obtained D’ = 80, H’ = 1600, W’ = 1408. In addition, we set T = 35 as the maximum number of random sampling points in each nonempty voxel. Two fully convolutional network (FCN) layers, FCN-1 (7, 32) and FCN-2 (32, 128). The final FCN mapped the output of VFE-2 to R128. Subsequently, feature extraction between point clouds was achieved using cubic convolutional fusion.

#### 5.4.3. Multimodal Fusion

Two 128-dimensional FCN modules were used to extract features from the point cloud. These modules extracted features after fusion, projecting image features onto each corresponding point. Then, 3 layers of conv3D convolution were used to perform voxel fusion of the information obtained after the point fusion. Finally, the Regional Suggestion Network (RPN) structure outputted the final result.

### 5.5. DAIR-V2X Dataset Evaluation

**Figure 7:**
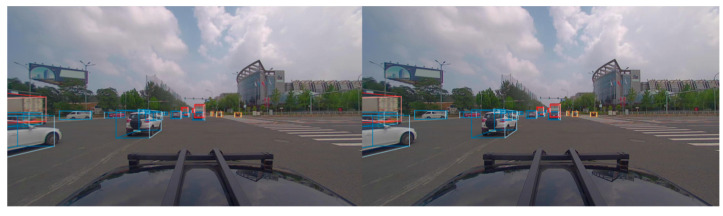
Diagram of algorithm test results.

This paper evaluated the detection performance using standard DAIR-V2X evaluation protocols (vehicle IOU = 0.7 and IOU = 0.5 and bicycle and pedestrian IOU = 0.5 and IOU = 0.25). Table 1, Table 2, Table 3 and Table 4 compare the algorithm presented in this article with the AP of commonly used algorithms in the 3D view and the BEV view. The results show that the three-dimensional target detection algorithm proposed in this paper significantly improves the detection performance compared with the commonly used algorithms. It is worth noting that the fusion effect is more pronounced in the 3D view score than in the BEV view scoring. It is worth mentioning that the proposed fusion technique outperforms the original voxel network through more powerful RPNs and is enhanced with additional data. In addition, this method has consistently outperformed the best-performing process in recent times. A sample test result of the proposed method is shown in Figure 7.

### 5.6. Performance Analysis of the AP Value Algorithm

As seen from Table 1, Table 2, Table 3 and Table 4, the algorithm proposed in this paper is the most advanced in detecting vehicles, pedestrians, and cyclists. It is better than the SECOND algorithm of single mode and the MV3D algorithm of multimode to a large extent. Especially in detecting small target pedestrians and cyclists in easy, medium, and difficult modes, the AP performance index of the algorithm proposed in this paper is improved by more than 7%. At the same time, in the case of IOU = 0.5, our algorithm can reach more than 60% in the BEV view and more than 50% in the 3D view. This is because the texture and color information provided by images in detecting small targets in this paper can compensate for the sparse point cloud. In vehicle detection, the AP performance index of the proposed algorithm can approach 80% when IOU = 0.7.

### 5.7. Stability Analysis

The algorithm’s stability is represented by calculating the mAP value of each method in this paper. A relatively high mAP value indicates better performance in detecting vehicles, pedestrians, and bicyclists, making the algorithm suitable for multisize, multitarget detections. Conversely, a low mAP value suggests that the algorithm is less effective in detecting one of the objects in the target detection. In Table 5, it can be observed that the proposed algorithm in this paper exhibits a 7.9% improvement in the mAP value in the BEV view and a 7.8% improvement in the 3D view compared to the Part-A2 network, which is an exceptional performer among the compared algorithms, with an IOU of 0.5 for cars and 0.25 for pedestrians and bicycles. Additionally, Table 6 demonstrates that when the IOU is set to 0.7 for cars and 0.5 for pedestrians and bicycles, the proposed algorithm shows a 5.4% improvement in the mAP value in the BEV view and a 4.3% improvement in the 3D view, compared to the SECOND algorithm, which is another remarkable performer among the compared algorithms.

### 5.8. Ablation Experiment

In order to explore the performance of the algorithm in a complex environment, we collected images and LiDAR data on rainy, foggy, and cloudy days in the DAIR-V2X dataset for model experiment verification. The experimental results are shown in Table 7 and Table 8.

As can be seen from Table 7 and Table 8, the proposed algorithm performs best in rainy, cloudy, and foggy environments in complex environments. Compared with conventional environments, the mAP performance of the BEV and 3D view decreases by 2% when IOU = 0.5 (vehicle) and IOU = 0.25 (pedestrian and bicycle). With an IOU of 0.7 (vehicle) and 0.5 (pedestrian and bicycle), the mAP performance of the BEV view and 3D view decreases by 3%, so we can see that the performance of our proposed algorithm is not significantly reduced in complex environments compared to other algorithms.

At the same time, to evaluate the camera’s performance under different viewing angles, the model we established was analyzed experimentally on the KITTI dataset, and the experimental results are shown in Table 9.

It can be seen from Table 9 that the algorithm we proposed also has the best performance on the KITTI dataset. The mAP value of the algorithm Part-A2 with a better performance than that of a single mode is increased by 1.27% and that of the MV3D algorithm with a multimode fusion framework is increased by 5.3%. The performance of our proposed algorithm on different datasets is relatively stable.

In order to consider the complexity and real-time performance, we tested each algorithm’s running time on a device equipped with an NVIDIA RX3060 graphics card and 12GB GPU memory. The experimental results are shown in Table 10.

From Table 10, the running time of the algorithm we proposed on the same device is not very dominant. However, the time is close to the existing multimode fusion algorithm, and no significant difference exists. This is related to the complexity of the model and the running time cost increases when the algorithm performance improves. In future research, we should continue exploring more efficient algorithms with the premise that the operating cost is unchanged.

## 6. Conclusions

The proposal presented in this paper is a multimodal fusion with multiple attention mechanisms for the 3D Target Detection Algorithm aimed at addressing the limitations of single-modal target detection. The first step involved utilizing the ResNet50 + FPN network framework to extract image features, resulting in the extraction of four-level features. Simultaneously, the point cloud feature extraction employed the voxel grid method and FCN to extract point and voxel features from each voxel. These extracted features were then considered the final features of the point cloud. Following this, regional point fusion and voxel fusion techniques combined the image and point cloud features. Once the fusion process was completed, the fused features underwent depth extraction using the SECOND network. Furthermore, the Coordinate attention mechanism and the SimAM attention mechanism were implemented during this stage. Finally, the RPN was applied to obtain the output. To validate and compare the proposed algorithm with other state-of-the-art algorithms, it was tested on the DAIR-V2X dataset. The results demonstrate that the proposed algorithm surpasses other algorithms regarding detection performance. However, due to the limitation of the 3D object detection dataset, the 3D object detection algorithm could not be studied under more severe conditions in this study. Therefore, in future research, datasets under harsh conditions will be collected by actual vehicles, types and scenarios of datasets will be enriched, and deep learning models based on Transformer will be built to improve the performance of 3D target detection.

## Figures and Tables

**Table 1:** Performance comparison of AP value algorithms with IOU = 0.5 and IOU = 0.25 from the BEV perspective.

Method	Car			Pedestrian			Cyclist		
	IOU = 0.5			IOU = 0.25			IOU = 0.25		
	Easy	Med	Hard	Easy	Med	Hard	Easy	Med	Hard
SECOND [49]	85.5	86.1	83.9	73.3	64.1	63.6	70.3	65.8	64.3
PointPillars [3]	85.5	83.9	83.7	66.9	55.3	54.8	62.8	59.8	58.7
Part-A2 [20]	85.1	85.4	83.4	65.9	59.8	59.3	66.6	61.7	60.5
3D-SSD [50]	84.2	83.9	82.0	N/A	N/A	N/A	N/A	N/A	N/A
MV3D [1]	85.4	84.2	83.1	74.2	65.0	63.3	69.4	64.5	63.2
PointPaint [51]	85.0	83.6	82.0	73.3	64.5	62.7	68.6	63.6	62.1
ContFusion [2]	84.8	83.1	81.4	72.8	63.5	61.6	67.2	62.2	60.8
Ours	88.4	86.6	86.5	81.5	71.0	70.7	71.6	70.8	70.9

**Table 2:** Performance comparison of AP value algorithms with IOU = 0.7 and IOU = 0.5 from the BEV perspective.

Method	Car			Pedestrian			Cyclist		
	IOU = 0.5			IOU = 0.25			IOU = 0.25		
	Easy	Med	Hard	Easy	Med	Hard	Easy	Med	Hard
SECOND [49]	81.9	83.0	83.6	57.5	49.3	48.9	65.0	59.6	58.7
PointPillars [3]	82.0	80.9	78.4	57.7	46.3	45.8	50.5	55.1	53.4
Part-A2 [20]	80.2	80.7	79.7	55.7	47.6	47.1	62.8	56.1	55.2
3D-SSD [50]	79.3	79.4	79.5	N/A	N/A	N/A	N/A	N/A	N/A
MV3D [1]	82.6	82.1	81.8	64.2	53.9	53.2	64.8	57.2	56.8
PointPaint [51]	81.4	81.0	80.5	62.6	51.4	50.6	63.7	56.3	55.4
ContFusion [2]	80.9	80.5	79.8	61.9	51.7	50.2	64.1	55.8	54.2
Ours	84.8	83.5	83.8	70.2	60.2	59.9	66.0	64.4	63.1

**Table 3:** Performance comparison of AP value algorithms with IOU = 0.5 and IOU = 0.25 from the 3D perspective.

Method	Car			Pedestrian			Cyclist		
	IOU = 0.5			IOU = 0.25			IOU = 0.25		
	Easy	Med	Hard	Easy	Med	Hard	Easy	Med	Hard
SECOND [49]	82.9	83.6	83.3	72.9	63.6	62.4	70.2	65.6	64.3
PointPillars [3]	83.0	83.5	81.1	66.7	55.0	54.1	62.7	59.8	57.9
Part-A2 [20]	84.2	83.2	82.4	65.5	59.5	59.0	65.9	60.9	60.2
3D-SSD [50]	82.3	81.9	72.5	N/A	N/A	N/A	N/A	N/A	N/A
MV3D [1]	85.4	85.6	83.1	74.3	64.1	63.8	69.2	64.8	63.9
PointPaint [51]	84.6	84.2	83.6	72.8	61.5	60.2	67.8	62.6	61.7
ContFusion [2]	82.7	82.6	82.1	71.3	60.4	59.6	66.1	61.5	60.2
Ours	87.8	86.2	83.8	81.1	70.6	70.3	70.5	70.6	70.7

**Table 4:** Performance comparison of AP value algorithms with IOU = 0.7 and IOU = 0.5 from the 3D perspective.

Method	Car			Pedestrian			Cyclist		
	IOU = 0.5			IOU = 0.25			IOU = 0.25		
	Easy	Med	Hard	Easy	Med	Hard	Easy	Med	Hard
SECOND [49]	75.8	73.9	70.9	52.8	43.4	42.9	63.2	56.5	55.7
PointPillars [3]	75.6	73.6	70.8	52.5	40.6	40.1	56.5	50.8	49.0
Part-A2 [20]	77.1	74.7	71.5	52.8	41.8	41.5	61.6	54.0	52.4
3D-SSD [50]	76.1	71.0	65.7	N/A	N/A	N/A	N/A	N/A	N/A
MV3D [1]	76.8	74.2	71.9	53.4	44.1	42.4	65.0	57.2	55.8
PointPaint [51]	75.9	75.2	71.4	52.8	42.7	40.8	63.8	55.7	54.2
ContFusion [2]	75.3	74.8	74.2	53.5	41.4	42.6	62.5	54.3	53.4
Ours	79.9	76.1	73.5	62.9	51.7	51.4	63.4	58.7	57.6

**Table 5:** mAP results at IOU = 0.5 and IOU = 0.25.

Method	IOU = 0.5 (Car) IOU = 0.25 (Pedestrian, Cyclist)	
	BEV	3D
SECOND [49]	66.0	72.1
PointPillars [3]	67.9	67.1
Part-A2 [20]	69.7	69.0
MV3D [1]	72.1	73.4
PointPaint [51]	71.4	70.8
ContFusion [2]	70.1	69.4
Ours	77.6	76.8

**Table 6:** mAP results at IOU = 0.7 and IOU = 0.5.

Method	IOU = 0.7 (Car) IOU = 0.5 (Pedestrian, Cyclist)	
	BEV	3D
SECOND [49]	65.3	59.4
PointPillars [3]	61.1	56.6
Part-A2 [20]	62.9	58.6
MV3D [1]	66.7	611
PointPaint [51]	65.4	62.1
ContFusion [2]	65.8	60.0
Ours	70.7	63.9

**Table 7:** mAP results in complex environments are IOU = 0.5 and IOU = 0.25.

Method	IOU = 0.5 (Car) IOU = 0.25 (Pedestrian, Cyclist)	
	BEV	3D
SECOND [49]	61.0	69.1
PointPillars [3]	62.9	62.1
Part-A2 [20]	66.7	66.0
MV3D [1]	67.2	69.6
PointPaint [51]	66.3	66.8
ContFusion [2]	64.5	63.2
Ours	75.6	74.8

**Table 8:** mAP results in complex environments are IOU = 0.7 and IOU = 0.5.

Method	IOU = 0.7 (Car) IOU = 0.5 (Pedestrian, Cyclist)	
	BEV	3D
SECOND [49]	55.3	47.4
PointPillars [3]	52.1	49.6
Part-A2 [20]	58.9	54.6
MV3D [1]	61.1	54.8
PointPaint [51]	60.2	53.2
ContFusion [2]	58.2	52.0
Ours	67.7	60.9

**Table 9:** mAP results on KITTI dataset.

Method	mAP
SECOND [49]	65.3
PointPillars [3]	64.07
Part-A2 [20]	68.33
MV3D [1]	64.3
PointPaint [51]	61.3
ContFusion [2]	60.2
Ours	69.6

**Table 10:** Comparison of the running time of the algorithm.

Method	Time (s)
SECOND [49]	0.046
PointPillars [3]	0.27
Part-A2 [20]	0.52
MV3D [1]	0.33
PointPaint [51]	0.31
ContFusion [2]	0.28
Ours	0.39

## Data Availability

The data in this paper is from DAIR-V2X’s Cooperative Vehicle Infrastructure3D Detection: https://thudair.baai.ac.cn/task-cooptest (accessed on 18 October 2023).
